# Comparing the effect of two types of silver nano-crystalline dressings (acticoat and agcoat) in the treatment of full thickness burn wound

**Published:** 2018-12

**Authors:** Faranak Alinejad, Mahnoush Momeni, Mohammad Javad Fatemi, Mostafa Dahmardehei, Soheila Naderi, Mohammad Reza Akhoondinasab, Masoume Zayedly, Omid Mahboubi, Hossein Rahbar

**Affiliations:** 1 Department of Infectious Diseases, Burn Research Center, Iran University of Medical Sciences, Tehran, Iran; 2 Department of General Surgery, Burn Research Center, Iran University of Medical Sciences, Tehran, Iran; 3 Department of Plastic Surgery, Burn Research Center, Iran University of Medical Sciences, Tehran, Iran; 4 Department of Marine Biology, Sciences and Research Branch, Islamic Azad University, Tehran, Iran; 5 Registered Nurse, Burn Research Center, Iran University of Medical Sciences, Tehran, Iran; 6 Life Sciences Student, Mac Master University, Hamilton, Canada; 7 Pediatrician, Burn Research Center, Iran University of Medical Sciences, Tehran, Iran

**Keywords:** Burns, Infection, Silver nano-crystalline dressing

## Abstract

**Background and Objectives:**

This study was conducted to compare the effect of acticoat and agcoat dressing (2 types of silver nano-crystalline dressings) in the treatment of burn wounds. Infection is one of the most important causes of death in patients with major burn. Despite using different prevention methods, including prophylaxis antibiotics with broad-spectrum antibiotics, no method has been found to prevent this dangerous complication for burn patients. Topical silver sulfadiazine is one of the best topical antibiotics in infection control of burn wounds, and other forms of AG dressings are also useful. Their advantages are slow releasing, further-half-life, less frequent dressing change, and less pain during replacement.

**Materials and Methods:**

In this study, 30 patients with infected full thickness burn wound were selected. The patients' age range was 18–85 years, with the mean age of 39.7–17.27. Every patient's wound was divided into 2 parts randomly, one part was dressed with agcoat and the other with acticoat. Sampling of the 2 parts was done before dressing and after the third and seventh day of dressing.

**Results:**

The positive outcome of the first day culturing before silver dressing was 80% and 76.7% for agcoat and acticoat, respectively. However, on the third day, it decreased to 30% and 33.3%, respectively. On the seventh day, it further decreased to 20% in both groups, and the percentage of bacterial growth reduction was not significant.

**Conclusion:**

Based on the results of this study, silver agcoat dressing was as effective as acticoat dressing in preventing burn wound infection.

## INTRODUCTION

Burns and thermal injuries are physically and psychologically destructive and potentially fatal. They are some of the most important medical emergencies and are considered to be one of the major causes of fatality and disability worldwide (1, 2).

Annually, about 725000 burn incidents occur in Iran. Despite receiving medical attention, a significant number of these people die and many others face permanent complications (3).

Studies in other countries have revealed that burns cause is an important factor for the patients' prognosis. For example, a study done in Turkey reported that electrical burns were the most prevalent causes of burns, with a lower fatality rate, while flame burns were the most deadly (4). On the other hand, in most studies, burns with hot liquids were the most prevalent reason of burning (5–9).

Different results were reported in studies conducted in Iran with regards to epidemiology and the relationship between burn cause and its consequence. In a study by Maghsoudi et al. in Tabriz, Iran, which was done on children younger than 14 years, flame burns were found to be the most prevalent cause of burns, with 6.4% fatality rate.

Burns with boiling water were common among children younger than 5 years old, and burning with flames were more common in other groups. In this study, the average burn was 19% and the fatality rate was 6.4%. In another study done in Shiraz, Iran, burns with hot liquids were the most common followed by flame burns (5).

Despite the new developments in controlling and treating burn wounds and provision of intensive care for these patients, infection is the main element of fatality among them (10, 11). One of the fundamental problems in each burn wound is the control of wound infection. A high percentage of immobility of different body parts on the one hand and existence of infection's factors on the other are the most common factors leading to death of burn patients (12).

Infection prevention and recognition of the cause lead to reduced fatality rate and reduction in complications for widespread burn (13). In developed countries, changing care methods and separation of patients had an important role in the reduction of infectious complications. (14). Since there are diverse types of infection in different burn departments, so periodic studies are particularly important in determining prevalent microorganisms (14).

The burn site is severely exposed to infection because of destruction of protective tissues. Various methods have been applied to prevent the infection of the burn site. Using prophylaxis antibiotic with widespread antibiotic was one of these methods. However, the prophylactic antibiotics are not successful in preventing burn site infection (15). Thus, the use of dressings that contained antibiotics or other compounds which could decrease or stop microbial growth environment were considered.

In some studies, the use of silver compounds was evaluated and considerable positive impacts of this element in prevention of burn site infection have been reported (16–18). Ionized silver dressings have different benefits as compared to creams containing silver sulfadiazine, including slow releasing, further-half-life, less frequent dressing change and thus less pain during replacement, and low rate of wound infection (17). Acticoat, which is available in Iran pharmaceutical market, is pricey, so it is not possible to use it for widespread burns. However, a new dressing containing silver called agcoat has entered Iran's pharmaceutical market. This study was conducted to compare the effect of acticoat (a domestic product) and agcoat (an imported product) on treating burn wounds.

## MATERIALS AND METHODS

Agcoat and acticoat dressings are different in terms of fabrication. Acticoat dressing is manufactured by chemical vapor deposition method (CVD). In this method, silver gas will be condensed into a vapor solution on a nylon surface. Fabrication method of Agcoat dressing is chemical wet deposition (CWD), a method in which the ion particles are put into a wet solution and then placed onto a nylon surface. The amount of silver in an agcoat dressing is between 0.3–0.5 mg in each *cm*^2^ which is significantly less compared to silver amount of acticoat dressing (0.69–1.64 mg by *cm*^2^).

This clinical trial was done in 2015 and 2016 in our hospital. Participants in this study were the patients with full thickness burns with various causes who had come to the center for treatment. Criteria for entry in the study were as follow: full thickness infected burns; absence of systematic diseases, such as diabetes; no history of other burns at the current site; individual informed consent for taking part in the study. Also, those who were reluctant to participate in the study were excluded.

A total of 32 burn patients were selected and the aim of research was explained to them as well as the types of dressings that would be used. A written consent form was signed by the patients. The wound infection was documented based on clinical and laboratorial confirmation and opinions of a skillful burn specialist and infectious disease specialist. Two patients dropped out.

**Fig. 1.**
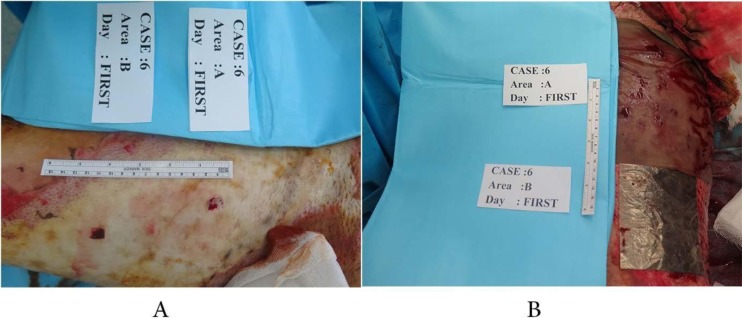
A case of full thickness burn wound which is divided in two parts randomlya) The preoperative photographb) The preoperative design: Ag and Acti coat

Each patient's infectious wound was divided into 2 equal parts, one was treated by acticoat dressing and the other by agcoat (Fig. 1). The infections were dressed simultaneously under sterile conditions, achieved by washing and disinfecting the burn site according to hospital protocols. Treatment was done in the same way for all patients, covering the whole wound surface, 30 with acticoat and 30 with agcoat dressings.

Wound dressings for all patients were performed in such a way that a gauze covered the whole burn, and 2 layers of gauze were used in each dressing. After applying the gauze containing silver on the wound, a simple sterile pad soaked in distilled water was put on it and then the whole wound was covered.

The wound dressing was changed after the third and seventh days. Before reapplying the dressing on the third and seventh day, the sampling was done from the wound and sent to the laboratory for culturing. The samples were cultured in blood agar, EMD, and chocolate agar. The outcome of culturing was compared between the 2 types of dressings. The rate of wound healing was compared based on the skin graft estimation.

The gathered information was evaluated using student's t test, chi-square, and Mann-Whitney test in SPSS version 24. Also, variance analysis test was done by repetition of observation.

## RESULTS

In this study, 32 burn patients were evaluated, but 2 were excluded from the study. The first patient was excluded due to dissatisfaction with continuing the treatment and the second because of their will to leave the hospital, and eventually 30 patients were studied. Patients' age range was 18–85 years, with the mean age of 39.7–17.27. Of the patients, 18 (60%) were men and 12 (40%) were women.

The distribution of patients' demographic variables is presented in Table 1.

**Table 1.** The distribution of patients' demographic variables

**Variables**		
Age	Mean	39/7 ± 17/27
	Below 30 years	8 (26/7%)
	30–39	10 (33/3%)
	40 Years or more	12 (40%)
Gender	Male	18 (60%)
	Female	12 (40%)
The average level TBSA		42/17 ± 18/21
Damage Cause	Explosion	8 (26/7%)
	Fire	13 (43/3%)
	Vapor	5 (16/7%)
	Contact	1 (1/7%)
	Electricity	3 (10%)

The frequency distribution of burn cause is shown by age and gender in Table 2. According to the results, fire, with the frequency of 9 of 18 (50%) was the most common cause of burns in men and flame and boiling water, with the frequency of 4 for each, were (33,3%) the most common among women. According to exact Fisher test, there was no significant difference between men and women in burn cause (p=0.19). The mean age of burn patients by explosion, fire, boiling water, contact and electricity was, respectively, 40.5 15.86, 34.92 10.89, 35.4 22.73, 85 and 50.33 17.62. Also, according to one-way analysis of variance, there was a significant difference between burn cause (p=0.039) and patients' mean age. However, there was no significant difference between burn cause and age group (p=0.29).

**Table 2.** Frequency distribution of burn cause in terms of age and gender

**Variables**		**Damage Cause**					**P**

		**Explosion**	**Fire**	**Vapor**	**Contact**	**Electricity**	
Gender	Male	6 (33/3)	9 (50)	1 (5/6)	1 (5/6)	1 (5/6)	0/19
	Female	2 (16/7)	4 (33/3)	4 (33/3)	0 (0)	2 (16/7)	
Mean		40/5 ± 15/86	34/92 ± 10/89	35/4 ± 22/73	85	50/33 ± 17/62	0/039
Age	Below 30 Years	2 (25)	3 (37/5)	3 (37/5)	0 (0)	0 (0)	
	30–39	2 (20)	7 (70)	0 (0)	0 (0)	1 (10)	0/29
	40 Years Or More	4 (33/3)	3 (25)	2 (16/7)	1 (8/3)	2 (16/7)	

Every patient's wound was divided into 2 parts, one part was treated with agcoat and the other with acticoat dressings. Sampling of the 2 parts was done before dressing and after the third and seventh day of dressing and was cultured *in vitro*.

**Diagram 1.**
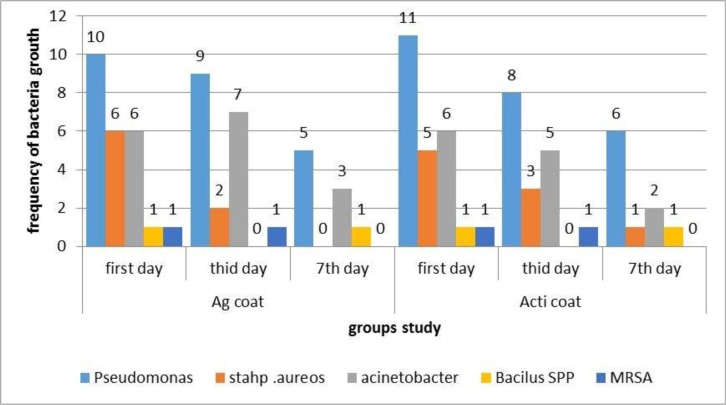
Frequency distribution of the bacteria's growth from the first to seventh days in both group

The culturing of the taken samples before testing the wounds showed the bacterial growth of 24 samples of agcoat group and 23 samples of acticoat group (80% vs 76.7%). However, according to the results of chi-square test, there was no significant difference between 2 groups (p=0.75). The frequency of bacterial growth of the taken samples on the third day in both groups of agcoat and acticoat was 19 and 18, respectively (63.3% vs 60%) and, again, there was no significant difference between the groups (p=0.79). Frequency distribution of microbial growth on the seventh day in both groups of agcoat and acticoat was 9 and 10 (30% vs 33.3%), respectively, and the difference between the 2 groups was not significant (p=78%) (Table 3). The frequency distribution of growing bacteria type on the first, third, and seventh days *in vitro* is demonstrated in Graph 4. According to the chart, *Pseudomonas* growth in both group decreased on the third and seventh days compared to the first day, while *Acinetobacter* growth decreased in acticoat but not inagcoat. *Staphylococcus aureus* growth decreased in both groups, however, overall, the frequency distribution of growing bacteria in the 2 groups was not significantly different *in vitro* (Table 3, Diagram 1).

**Table 3.** Frequency distribution of the bacterial growth from taken samples of the first, third and seventh

**Day**	**Bacteria's growth in two groups**		**P**

	**Agi coat**	**Acti coat**	
First	24 (80)	23 (76/7)	0.75
Third	19 (63/3)	18 (60)	0.79
Seventh	9 (30)	10 (33/3)	0.78

According to the results, from the 3 samples taken from each wound, in 14 samples of agcoat group and 15 acticoat group, bacteria were grown in just 1 sample. Also, out of 10 samples of agcoat and 9 samples of acticoat, 3 samples cultured and bacteria grew in 2 samples. In 6 samples of the 2 groups (3 in each), all 3 cultured samples were positive. At the same time, according to the results of chi-square test, there was no significant difference between the 2 groups in bacterial growth (p=0.96) (Table 4).

**Table 4.** Items of frequency distribution of the bacteria's growth from the first to seventh days in both group

**Variables**	**Group**		**P**

	**Agi coat**	**Acti coat**	
Total number of cultured samples	30 (100)	30 (100)	
Positivity of one out of three samples	14 (46/7)	15 (50)	
Positivity of 2 out of 3 samples	10 (33/3)	9 (30)	0.96
Positivity of all three cultured samples	6 (20)	6 (20)	

The success rate of skin graft of the 2 groups of agcoat and acticoat was 97.7 3.77 and 97 3.48, respectively, and there was no significant difference between the 2 group (p=0.46). Moreover, the success rate of agcoat group was optimal in 17 cases (less than 100%) and excellent in 12 cases (100%). In acticoat group, the success rate of 13 cases was optimal and of 18 cases was excellent. The success rate of frequency distribution in terms of drug and other characteristics of patients is displayed in Table 5. Also, there was no significant difference in the success rate of treatment in terms of age, type of dressing, material, and damage cause (Table 5).

**Table 5.** Success rate distribution in terms of treatment and other characteristics of patients

**Variables**		**Success amount**		**P**

		**Optimal**	**Perfect**	
Dressing type	Agi coat	17 (58.6)	12 (41.4)	0.2
	Acti coat	13 (41.9)	18 (58.1)	
Age group	Below 30 years	4 (50)	4 (50)	
	30–39	7 (70)	3 (30)	0.58
	40 Years or more	6 (50)	6 (50)	
Gender	Male	9 (50)	9 (50)	
	Female	8 (66.7)	4 (33.3)	0.37
Damage cause	Explosion	4 (50)	4 (50)	
	Fire	8 (61.5)	5 (38.5)	
	Vapor	3 (60)	2 (40)	0.79
	Contact	1 (100)	0 (0)	
	Electricity	1 (33.3)	2 (66.7)	

## DISCUSSION

Infection is one of the greatest causes of death in patients with widespread burn. Many studies have been done on how to prevent death, but despite the use of different prevention methods, including prophylaxis antibiotics with broad-spectrum antibiotics, no single method is proven entirely to prevent the dangerous complications in burn patients. On the other hand, some studies have shown that the local use of silver-containing compounds on wounds caused by burn can significantly prevent infection. In addition to the relative impact of silver-containing compounds of ionized silver dressing versus creams containing ionized silver, such as silver sulfadiazine cream, dressings have several advantages, such as slow releasing, longer half-life, less frequent dressing change and thus less pain during replacement, and low expectancy of infected wounds. In burn patients who cannot change the dressing themselves, ionized silver is superior to silver sulfadiazine cream (17). The price of acticoat is costly and in many cases, it is not possible to provide this type of dressing for the health care system and the patients.

The outcome of the first day of culturing, before silver dressing, in agcoat and acticoat was 80% and 76.7%, respectively. However, on the third day, it decreased to 30% and 33.3%, respectively, in the 2 groups, and it decreased to 20% in both groups on the seventh day; the percentage of bacterial growth reduction was not significant. Thus, based on the results of this study, dressing containing silver agcoat is as effective as acticoat in preventing burn wound infection. In a review done by Joe et al, silver dressing was introduced as a suitable method to prevent infection and success of bonded grafts in burn wounds site.

Type of grown bacteria in cultures on the first, third, and seventh days was significantly different in both groups. Also, *Pseudomonas* was the most common bacterium in both groups on the first, third, and seventh days, but its frequency was reduced each time. In a study by Bishara et al. (2007), use of silver-containing compound in chronic and open wounds caused by burning led to a sharp drop of various bacteria-especially *Acinetobacter* and *Pseudomonas*. Also, they found that this dressing on such wounds had an acceptable impact on wound healing acceleration and graft success (17). Their findings were similar to ours, as the success rate of graft in both domestic and foreign silver dressings had a significant effect on graft success and that there was no significant difference between the 2 in wound healing percentage. Nevertheless, previous studies have shown that among natural metals, silver ions have strong antimicrobial properties against many bacterial species (19, 20). Because of the super antimicrobial feature of silver and less toxicity of free ion body cells, Moyer used silver for the first time with density of 5% as silver nitrate to treat burns (21). Also, because of the increase of infectious diseases and unexpected increase of microorganism's resistance to antibiotics, most researchers aimed to find new methods for preventing this resistance. Fortunately, previous findings have encouraged researchers to renew their interest to silver compounds to prevent bacterial growth because bacteria can not develop resistance when treated by silver compounds (22). In this regard, the results of our study also showed that compounds containing silver have a lethal and inhibitory effect on the microorganisms that cause infection in burn patients and ultimately accelerate wound healing.

The results of this study revealed that burn wound dressings with silver compounds lead to the control of microbial growth and reduce wound infection. Also, these dressings accelerate skin graft and wound healing and help better control and treat wound infections. There were no significant differences between the effectiveness of agcoat and acticoat. Agcoat, which is available in the market, is effective to some extent in preventing wound infection caused by burn-similar to acticoat.

The different rates of improvement with each of the dressings and any possible complications resulting from their use should be investigated in future studies.
